# Screening and functional analysis of *StMYB* transcription factors in pigmented potato under low-temperature treatment

**DOI:** 10.1186/s12864-024-10059-x

**Published:** 2024-03-18

**Authors:** Bi-Cong Chen, Xiao-Jie Wu, Qiu-Ju Dong, Ji-Ping Xiao

**Affiliations:** College of Agronomy and Biotechnology, Yunnan Agricultural University, No.95 Jinhei Road, Panlong District, Kunming City, 650201 Yunnan China

**Keywords:** Pigmented potato, Low-temperature treatment, MYB transcription factor, Bioinformatics analysis, Anthocyanins

## Abstract

MYB transcription factors play an extremely important regulatory role in plant responses to stress and anthocyanin synthesis. Cloning of potato *StMYB*-related genes can provide a theoretical basis for the genetic improvement of pigmented potatoes. In this study, two MYB transcription factors, *StMYB113* and *StMYB308*, possibly related to anthocyanin synthesis, were screened under low-temperature conditions based on the low-temperature-responsive potato *StMYB* genes family analysis obtained by transcriptome sequencing. By analyzed the protein properties and promoters of *StMYB113* and *StMYB308* and their relative expression levels at different low-temperature treatment periods, it is speculated that *StMYB113* and *StMYB308* can be expressed in response to low temperature and can promote anthocyanin synthesis. The overexpression vectors of *StMYB113* and *StMYB308* were constructed for transient transformation tobacco. Color changes were observed, and the expression levels of the structural genes of tobacco anthocyanin synthesis were determined. The results showed that *StMYB113* lacking the complete MYB domain could not promote the accumulation of tobacco anthocyanins, while *StMYB308* could significantly promote the accumulation involved in tobacco anthocyanins. This study provides a theoretical reference for further study of the mechanism of *StMYB113* and *StMYB308* transcription factors in potato anthocyanin synthesis.

**Supplementary Information:**

The online version contains supplementary material available at 10.1186/s12864-024-10059-x.

## Introduction

Potato is one of the most important crops in the world and is widely cultivated because its tubers are rich in starch and nutrients [1]. Pigmented potato plants are commonly cultivated potato [2] that contain more polyphenolic compounds and higher antioxidant activity than ordinary potato plants [3, 4], especially when the polyphenolic natural pigment mixture is rich in anthocyanins, which have a positive impact on human health. Pigmented potatoes can be used as plant-derived materials for obtaining natural anthocyanins [5], and targeting anthocyanins as a target trait in breeding programs can ensure that varieties are bred to meet the nutritional needs of human consumption in developing countries [6].

Plants exhibit increased synthesis of polyphenols under abiotic stress conditions, which helps plants cope with environmental constraints. Anthocyanins are a class of flavonoids that are ultimately derived from phenylalanine [7], and flavonoid gene expression is tightly regulated by environmental and developmental signals [8]; phenylalanine biosynthesis is involved in this biosynthetic pathway. Activation under abiotic stress conditions [9] can lead to further accumulation of anthocyanins. Low-temperature stimulation is also an important condition for promoting anthocyanin synthesis [10]. It has been reported that low temperature can promote the synthesis of anthocyanins in red grape peels [11] and Mikania micrantha leaves [12], thereby improving the adaptability of plants to low-temperature environments.

In recent years, a large number of studies have shown that low temperature promotes the accumulation of anthocyanins by upregulating the expression of structural genes in the anthocyanin biosynthesis pathway. After low-temperature treatment, almost all genes directly involved in the late stage of anthocyanin biosynthesis exhibited high expression levels in *Brassica rapa* L. [13]; the expression levels of *CHS3* (Chalcone synthase 3), *F3’H1* (Flavonoid 3'-hydroxylase 1), *MYBA1(Myeloblastosis oncoprotein A1)*, and *UFGT* (3-O-Flavonoids Glucosyltransferase) significantly increased [14]; and when apples were induced by low temperature, transcription factors related to anthocyanin synthesis were significantly upregulated, increasing the content of anthocyanins [15]. The early anthocyanin biosynthetic structural genes EBGs (*SmCHI* (Chalcone isomerase), *SmF3H*) in eggplant were more sensitive to low temperature than the late biosynthetic structural genes LBGs (*SmF3′5’H* (Flavonoid 3'-hydroxylase), *SmDFR* (Dihydroflavonol-4-reductase) and *SmANS* (Anthocyanidin synthase)) [16]. Our sequencing results indicate that in the skin of colored potato tubers, *StF3'H*, *StF3′5'H*, *StDFR*, *StANS*, and *StUFGT* were highly expressed, and *StDFR* is upregulated in the flesh of potatoes. These findings indicate that low temperature may regulate the content of anthocyanins by regulating the expression of structural genes.

In addition, MYB transcription factors in the MBW complex can also be involved in regulating the accumulation of anthocyanins at low temperature; for example, apple *MdMYBPA1* initiates anthocyanin synthesis in red-fleshed apples at low temperature [17], and tomato *SlAN2* at low temperature can act as a positive regulator of anthocyanin synthesis in fruit [18].

The MYB family is one of the most important gene families involved in regulating plant growth and development and responding to abiotic stress [19]. The incompletely repeated and highly conserved sequence at the N-terminus of the MYB transcription factor is called the MYB domain [20]. According to the amino acid sequence and gene structure of the MYB domain, the MYB genes are divided into 1R-MYB, R2R3-MYB, R1R2R3-MYB and 4R-MYB protein families [21]. Research has suggested that the main regulators of anthocyanin biosynthesis are the encoding transcription factor R2R3-MYB, bHLH, WD40 and the MBW complex (MYB-bHLH-WD40) [8]. In the activation of R2R3-MYB transcription factors, most of the domains are located at the N-terminus, and the active domain or inhibitory domains are located at the C-terminus [22]. For example, R2R3-MYB transcription factors, including *AtMYB113* and *AtMYB114*, are involved in the anthocyanin biosynthesis pathway as positive regulators in Arabidopsis [23]; In apples, *MdMYB1*, *MdMYB10* has been shown to be responsible for the biological control of anthocyanin synthesis [24] [25]; AcMYBF110 in red-fleshed kiwifruit plays an important role in the regulation of anthocyanin accumulation by specifically activating the promoters of several anthocyanin pathway genes [26]. *StAN1* is thought to be involved in key rregulation of anthocyanin biosynthesis in potato leaves, the tuber epidermis and tubers [27]. Liu et al. rreported that *StMYBA1* and *StMYB113* promote anthocyanin biosynthesis in tobacco leaves [28]. Moreover, *StAN1* can activate the promoter activity of structural genes involved in potato anthocyanin synthesis, such as *StCHS*, *StCHI* and *StF3’H*, to promote the accumulation of anthocyanins in potato leaves [29].

Although the low-temperature promotion of anthocyanin biosynthesis has been studied in a variety of plants, studies on the synthesis and regulatory mechanism of anthocyanins in underground organs such as potatoes are rare. As the structural genes involved in anthocyanin synthesis in potato have been relatively well studied and their roles are limited, the tissue specificity [30] and evolutionary rate [31] of transcription factors involved in flavonoid pigment sympathy are greater than those of the structural genes they target and can regulate multiple structural genes to promote anthocyanin synthesis [32]. Through transcriptome sequencing, we screened two MYB-like transcription factors that were significantly expressed under low-temperature treatment, *StMYB113* and *StMYB308*, and found that their transcription levels were positively correlated with anthocyanin content, indicating that these transcription factors may be involved in regulating the biosynthesis of pigmented potato anthocyanins. This study provides a theoretical foundation for further analysis of the MYB transcription factors that regulate the synthesis of potato anthocyanins under low-temperature treatment.

## Results

### Analysis of the potato MYB genes family

#### Acquisition, chromosomal location and gene structure analysis of members of the potato MYB gene family

**Fig. 1:**
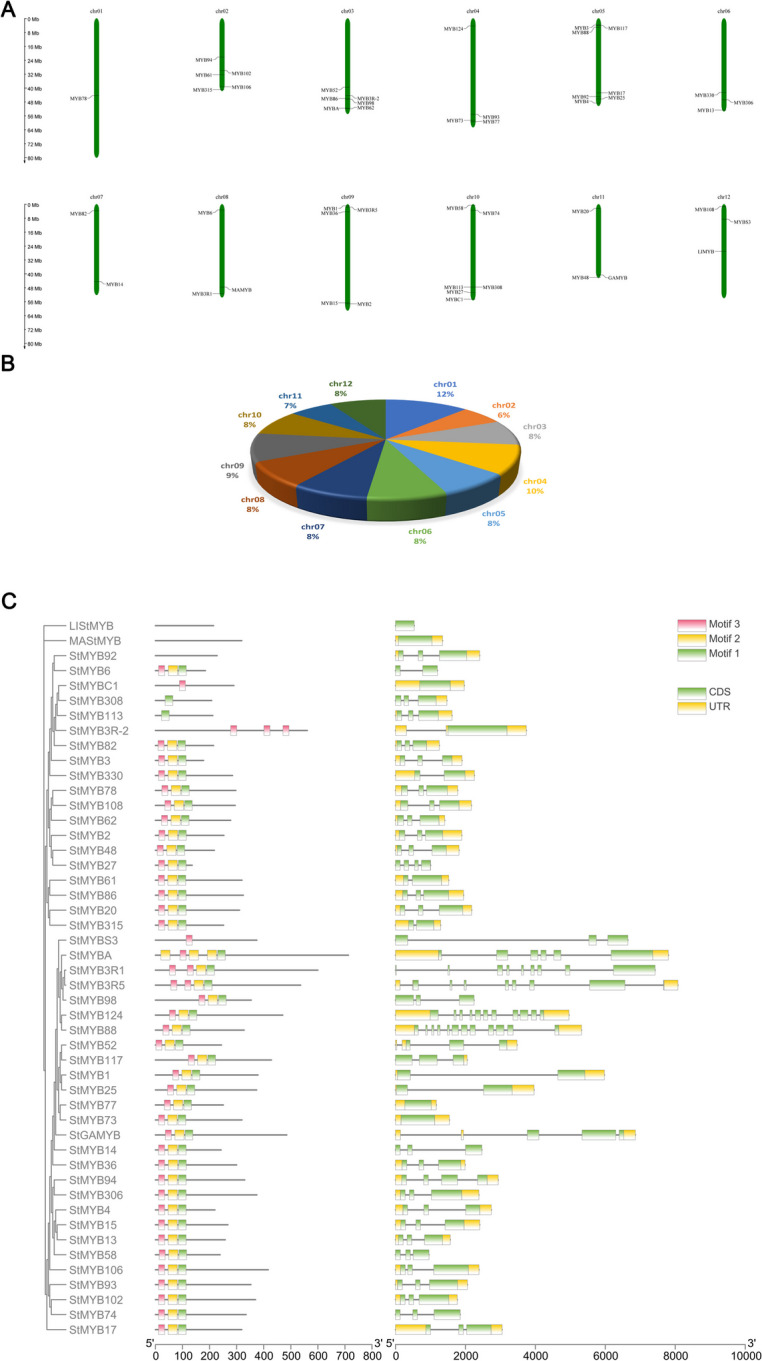
**A** Location of 48 *StMYB* genes on 12 chromosomes of potatoes. **B** The length of 48 *StMYB* genes on 12 chromosomes of potatoes. **C** Structural and conserved motif analysis of *StMYB* family genes

The *StMYB* sequences obtained by transcriptome sequencing were screened, the repetitive and erroneous sequences were removed manually. Finally, 48 *StMYB* transcription factors were obtained and named according to the transcriptome annotations. Chromosome localization analysis revealed the distributions of *StMYB* family members in different numbers and densities on twelve potato chromosomes (Fig. 1A). Among them, the number of *StMYB* genes distributed on chromosomes 5 and 10 were the largest, with a total of 10, followed by chromosomes 3 and 9, which contained 6 *StMYB* genes. Chromosome 1 had the lowest number of *StMYB* genes, with only 1 but the longest, while the shortest chromosome 2 had 5 *StMYB* genes (Fig. 1B). All *StMYB* genes were mapped to specific chromosomes.

To understand the gene structure characteristics of *StMYB* genes family members, we used the CDS of StMYBs and the corresponding amino acid sequences to analyze the gene structure of StMYBs (Fig. 1C). All 48 *StMYBs* had CDS coding sequences (Fig. 1C), and conservation motif prediction analysis revealed that there were 3 motifs in the potato StMYB protein sequences. We found that almost all StMYB protein sequences contained three conserved motifs, *StMYB113* clustered in the same subfamily only had motifs 1 and 2, and *StMYBC1* and *StMYBS3* had only motif 3. In addition, the corresponding positions of the motifs of each StMYB protein are relatively conserved.

### Evolutionary relationship of potato MYB genes

**Fig. 2:**
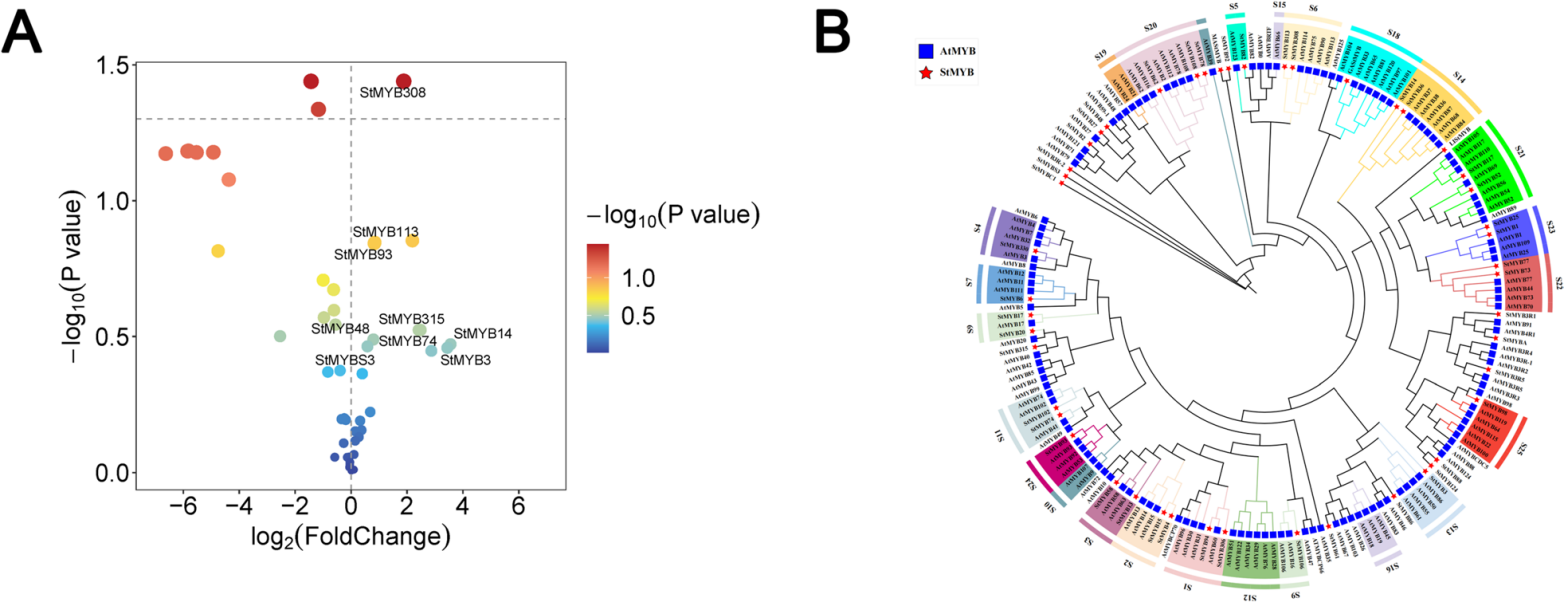
**A** Volcano plot of the low-temperature screening of *StMYBs*. **B** AtMYB and StMYB family phylogenetic tree analysis

As shown in Fig. 2A, 48 StMYBs exhibited differential gene expression between CK and low-temperature-treated samples. Among the 48 StMYBs, 18 were upregulated and 30 were downregulated. There were 9 genes upregulated more than 1.5 times fold change, and *StMYB308* was extremely significantly upregulated.

To investigate the evolutionary relationship and taxonomy of StMYB family members, a neighbor-joining evolutionary tree was constructed with the full-length protein sequences of 48 StMYBs and 123 Arabidopsis AtMYBs (Fig. 2B). The Arabidopsis MYB members are divided into 25 subfamilies. According to the classification of AtMYB proteins, the potato MYB (*StMYB*) genes obtained by sequencing were divided into 19 subfamilies, and the 10th, 12th, and 15th subfamilies in Arabidopsis were missed (corresponding to the 16th and 19th subfamilies of potatoes, respectively). The results of phylogenetic tree analysis showed that the *StMYB113* and *StMYB308* genes we cloned were clustered with the low temperature-responsive S18 family in *Arabidopsis thaliana* and in one branch with the S6 family, which is related to the regulation of anthocyanin synthesis in *Arabidopsis thaliana*. [33]; thus it is speculated that *StMYB113* and *StMYB308* are speculated to regulate the synthesis of potato anthocyanins in low-temperature environments.

### Bioinformatics analysis of *StMYB113* and *StMYB308*

#### Analysis of the protein properties of potato *StMYB113* and *StMYB308*

**Fig. 3:**
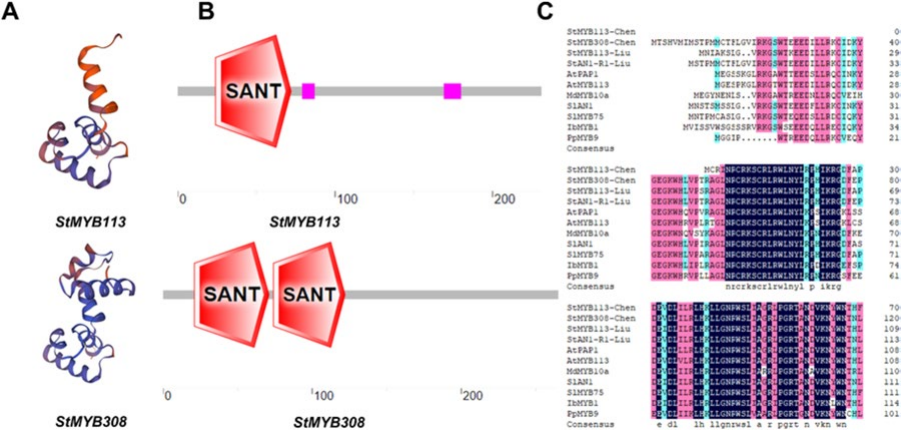
Analysis of *StMYB113* and *StMYB308* protein properties. **A **Protein tertiary structure prediction. **B** Protein domain analysis. **C** Multiple alignment of amino acid sequences

Through prediction analysis of the tertiary structures and domains of *StMYB113* and *StMYB308* (Fig. 3A, B), it was found that *StMYB113* has a SANT/MYB domain and is not a typical R2R3-MYB protein; *StMYB308* has two SANT/MYB domains and is a typical R2R3-MYB protein.

The protein sequences encoded by potato *StMYB113* and *StMYB308* were aligned with those of other species that regulate anthocyanin synthesis (Fig. 3C). Compared with *StMYB113*, which was found by Liu et al. to positively regulate anthocyanin synthesis, our *StMYB113* has only one typical conserved MYB domain at its N-terminus and is not an R2R3 MYB-type MYB transcription factor; *StMYB308* has two typical conserved MYB domains at its N-terminus. The conserved domain is highly similar to the protein sequence alignment of StAN1 [34], which has been proven to positively regulate anthocyanin function. Liu et al. reported that the 10 amino acids at the C-terminus of the *StMYB* transcription factor affect anthocyanin synthesis [34]. Both *StMYB113* and *StMYB308* have 10 complete amino acids, and it is predicted that they may function in regulating anthocyanin synthesis.

### Promoter analysis of *StMYB113* and *StMYB308*

**Fig. 4:**
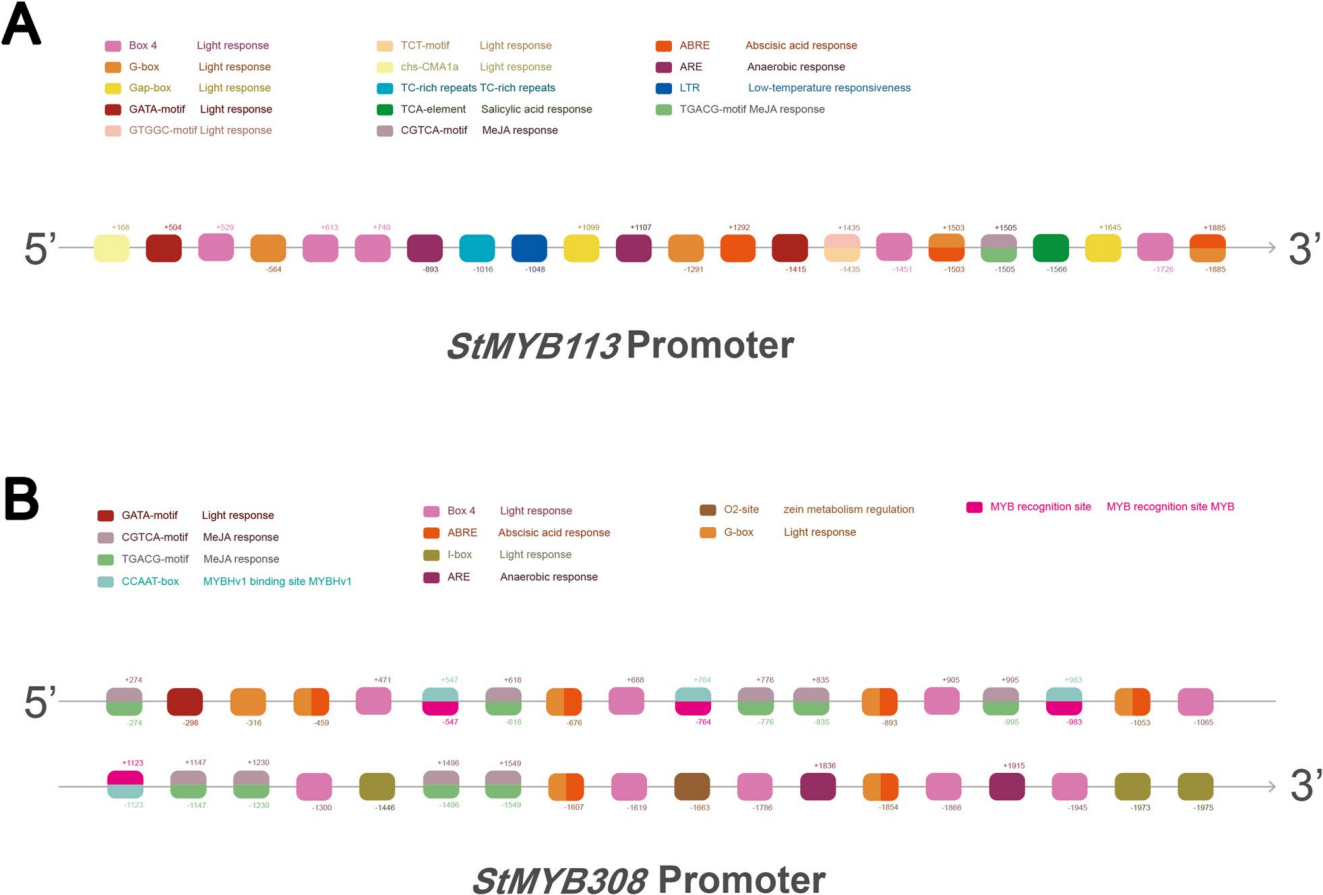
**A** *StMYB113* promoter homeopathic element prediction. **B** *StMYB308* promoter homeopathic element prediction

The promoter regions of *StMYB113* and *StMYB308* contain multiple response element sites (Fig. 4), such as G-box, Gap-box, Box and I-box related to light response, stress-related anaerobic response element ARE, series stress response, the element TC-rich repeats and the low-temperature response element LTR. Furthermore, *StMYB308* also has a MYB binding site.

#### Changes in structural gene expression levels in pigmented potato tubers treated with different temperatures

**Fig. 5:**
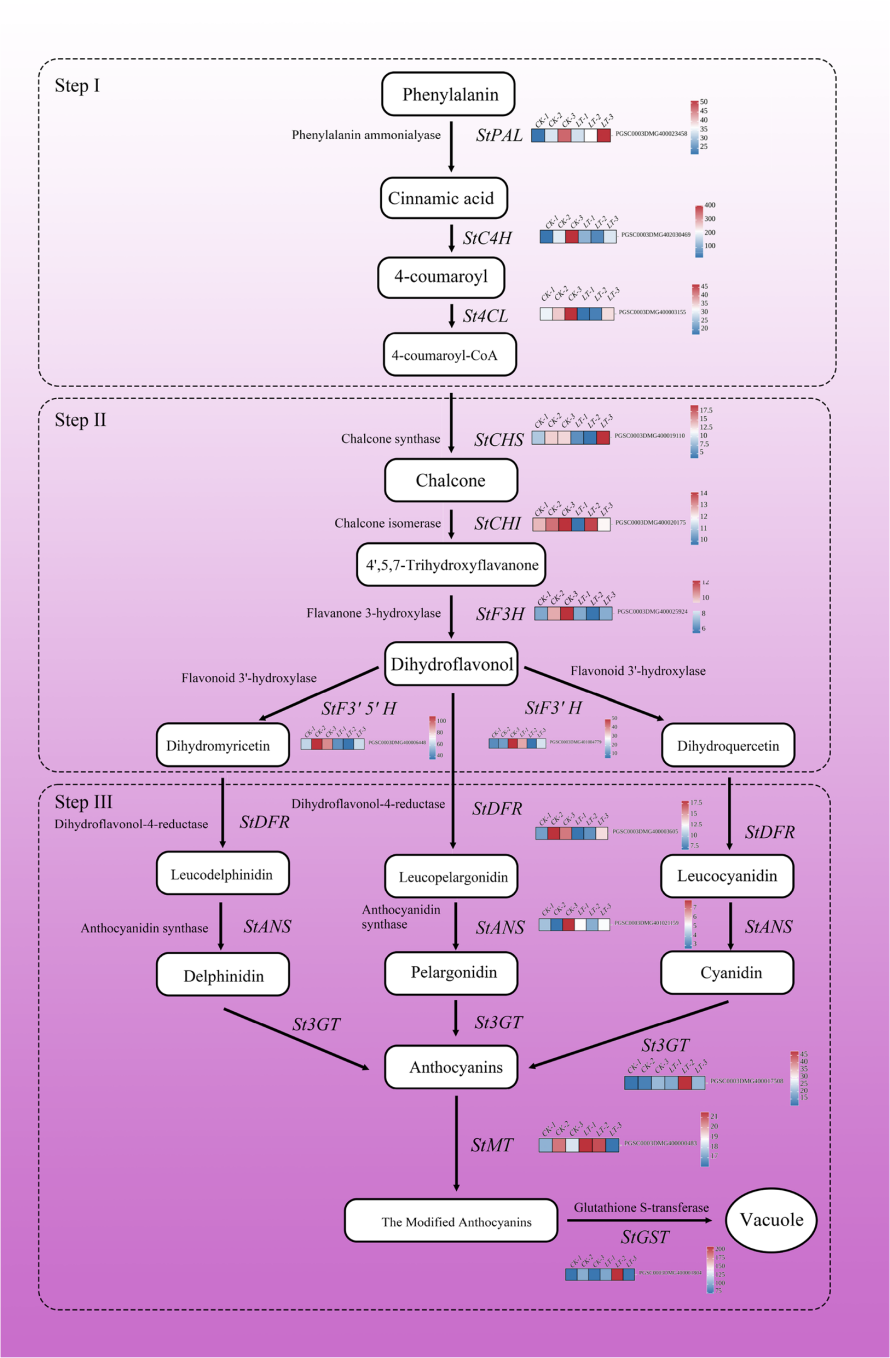
Heatmap of structural gene expression in the anthocyanin synthesis pathway. Red represents rising, blue represents falling. According to the Duncan test, different letters indicate a significant difference (*p* < 0.05) among the treatments. The error bars represent the mean ± SE)

To further explore the mechanism by which low temperature affects the regulation of anthocyanin synthesis, we analyzed the expression levels of related structural genes in the anthocyanin biosynthetic pathway. As shown in Fig. 5, most of the structural genes expressed in the tubers that were transcriptome sequenced showed a decreasing trend; however, structural genes that were expressed at the end of the synthetic pathway, such as *3GT* (Flavonoids 3-O-glycosyltransferase), *MT* (Methyltransferase), and *GST* (Glutathione S-transferase), showed an increasing trend. The reason for this result may be because the amount of anthocyanin accumulation in the tubers sent for sequencing peaked, and most of the structural genes promoted the metabolism of phenylpropanoids and provided essential compounds for the metabolism of flavonoids in the latter stage [35, 36], However, a significant increase in the expression of structural genes with end modified anthocyanins can prove that more anthocyanins are stably present in plants [37], indicating that low temperature can promote the progression of anthocyanin synthesis and the generation of more stable anthocyanins.

#### Differential expression of *StMYB113* and *StMYB308* genes in potato tubers at different stages and under different treatments

**Fig. 6:**
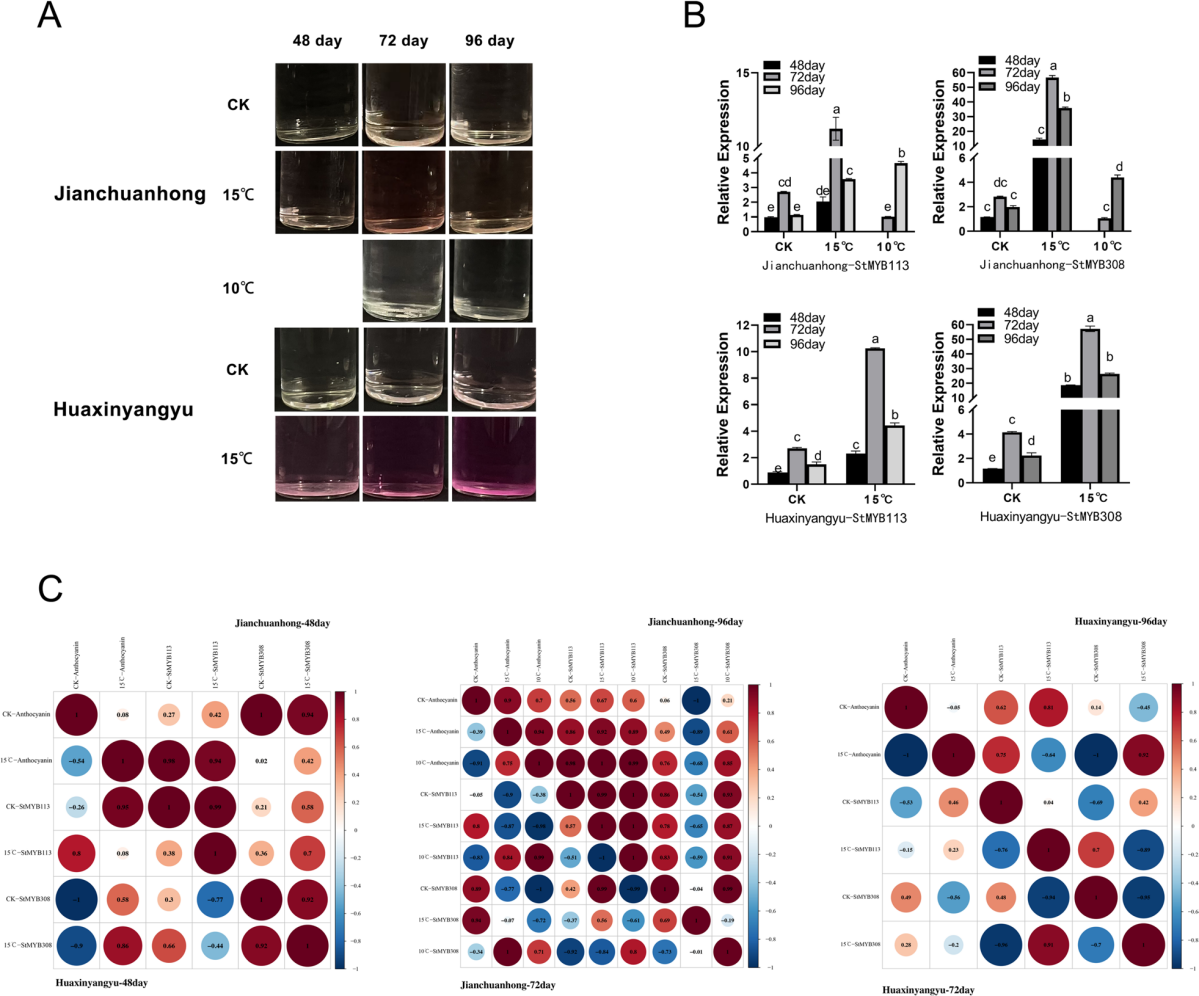
**A** Three temperature treatments of anthocyanin extracts from Jianchuanhong and Huaxinyangyu during three periods. **B** Expression patterns of potato *StMYB113* and *StMYB308* at different stages and treatments. **C**. Correlation analysis between anthocyanin content and *StMYB* genes expression

Compared with the color change caused by the anthocyanin extract at different times and under different treatments (Fig. 6A), which was used as a control, the expression pattern of the genes changed significantly under the low temperature treatment at 15°C, which was the same as the change in color change caused by the tuber anthocyanin extract (Fig. 6A, 6B). In Jianchuanhong tubers, the relative gene expression levels of *StMYB113* and *StMYB308* both 72 days > 96 days > 48 days under both the CK treatment and 15°C treatment; in tubers treated at 10°C, the gene expression levels were 96 days > 72 days. The expression levels were extremely low at 72 days, and the expression levels of the two genes increased suddenly at 96 days but were not higher than those at 15°C at 96 days. In Huaxinyangyu tubers, the expression levels of *StMYB113* and *StMYB308* were both 96 days > 72 days > 48 days in the CK and 15°C treatments. The relative expression levels of the *StMYB113* and *StMYB308* genes in the two pigmented potato tubers were upregulated during each period under 15°C, and the change trend was the same as that of the color change in the anthocyanin extract (Fig. 6A). Correlation analysis between the anthocyanin content and the expression levels of the *StMYB113* and *StMYB308* genes was carried out according to the treatment period (Fig. 6C), and the anthocyanin content in each period was positively correlated with the expression levels of the *StMYB113* and *StMYB308* genes.

### Prediction of protein interactions between *StMYB113* and *StMYB308*

**Fig. 7:**
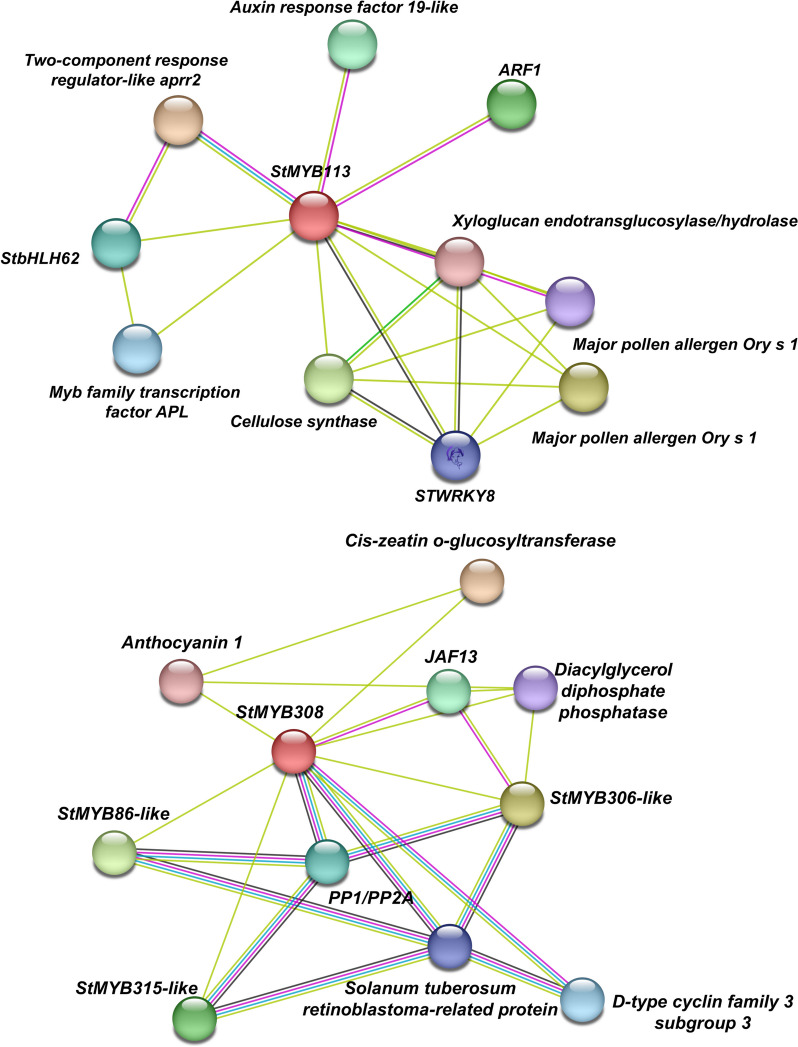
Prediction of protein interactions between *StMYB113* and *StMYB308*

The protein‒protein interaction prediction of the *StMYB113* and *StMYB308* genes were performed via the Search Tool for the Retrieval of Interacting Genes/Proteins (STRING) (https://cn.string-db.org/) website (Fig. 7). The results showed that *StMYB113* and *WRKY8* had an interaction relationship. WRKY8 plays a role in plant defense responses and participates in body regulation [38]; *StMYB308* interacts with *JAF13*. Several studies have shown that *StJAF13* is a bHLH transcription factor that can regulate the biological activity of anthocyanins by interacting with *StAN2* synthesis [39].

### Subcellular localization of *StMYB113* and *StMYB308* in potato

**Fig. 8:**
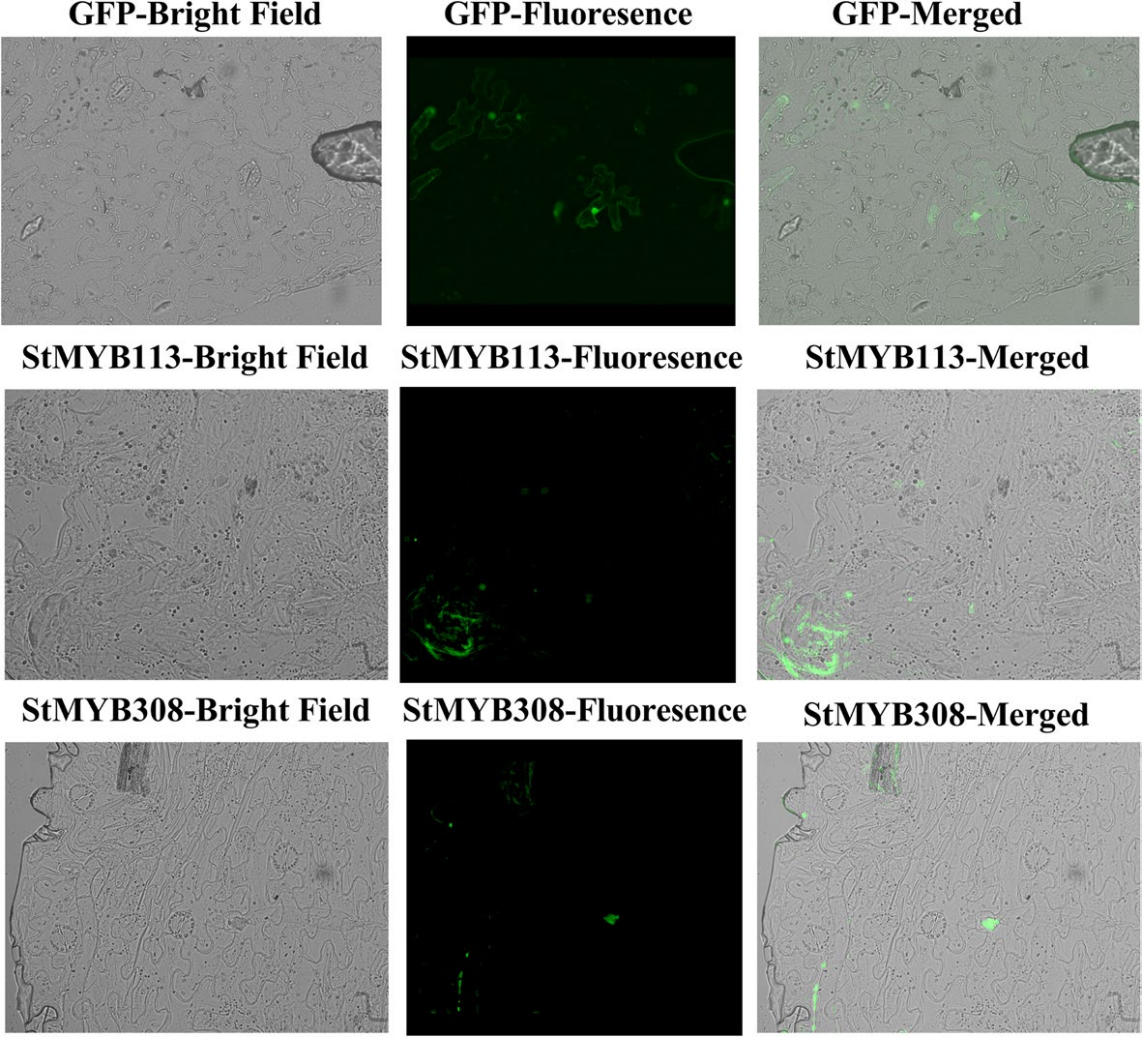
Subcellular localization of *StMYB113* and *StMYB308*

As shown in the Fig. 7, *N. benthamiana* epidermal cells transformed with *StMYB113*-YEP and *StMYB308*-YEP vectors exhibited green fluorescence signals colocalized with DAPI staining signals in the nucleus (Fig. 8), indicating that *StMYB113* and *StMYB308* are localized in the nucleus and function in the nucleus and have nuclear transcriptional activation activity.

### Color change of tobacco transiently transformed with *StMYB113* and *StMYB308*

**Fig. 9:**
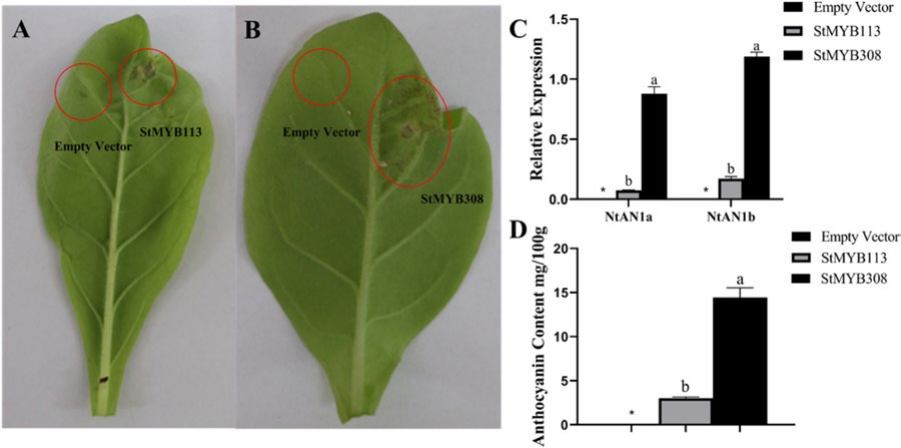
Phenotype, gene expression and anthocyanin content of transgenic tobacco **A**. *StMYB113* transgenic tobacco **B**. *StMYB308* transgenic tobacco **C**. Gene expression of *StMYB113* and *StMYB308* in transgenic tobacco **D**. Average anthocyanin content in *StMYB113, StMYB308* transgenic tobacco and those infected with the empty vector Duncan’s multiple comparisons were used for analysis (*P* < 0.05, *n* = 3)

To verify whether the *StMYB113* and *StMYB308* proteins can respond to low temperature and promote the accumulation of anthocyanins, we constructed the *StMYB113*-T and *StMYB308*-T overexpression vectors for transient transformation of tobacco. The results showed that tobacco injected with *StMYB113*-T didn’t cause phenotypic color changes, and the leaves didn’t exhibit purple spots, while the tobacco injected with *StMYB308*-T exhibited obvious purple spots and pigment accumulation in the leaves after transformation for ten days (Fig. 9A, B).

To further validate the promotion of anthocyanin accumulation in tobacco leaves by overexpression vectors, total RNA was extracted from leaves with purple spots, and cDNA was inverted. The qRT‒PCR results showed that, compared to those in leaves injected with no load, the expression levels of the endogenous transcription factors *NtAN1a* and *NtAN1b* related to anthocyanin synthesis in tobacco leaves injected with *StMYB308* significantly increased, while the expression levels of the transcription factors *NtAN1a* and *NtAN1b* in tobacco leaves injected with *StMYB113* significantly change. These results further confirmed that *StMYB308* promoted an increase in the tobacco anthocyanin content.

The content of anthocyanin in the leaves of the plants in the injection area of three transgenic tobacco lines, namely, *StMYB113* transgenic tobacco, *StMYB308* transgenic tobacco, and empty vector–treated tobacco, was measured. The anthocyanin content in the leaves of *StMYB308* transgenic tobacco was significantly higher than that in the leaves of plants in the empty vector control group. The anthocyanin content in the leaves of transgenic *StMYB113* transgenic tobacco did not significantly increase (Fig. 9 C, D), which was consistent with the phenotypic and qRT‒PCR results.

## Discussion

In potato, R2R3-MYB transcription factors are particularly important for regulating anthocyanin synthesis in different tissues [40], and the R2R3-MYB transcription factors *StAN1* [41], *StMYBA1* and *StMYB113* [28] have been shown to positively regulate anthocyanin synthesis, while transcription factors with incomplete R2R3-MYB domains cannot promote anthocyanin accumulation [34].

Low temperature can affect pigment accumulation by activating the MYB transcription factor [42], which is an important condition for promoting anthocyanin synthesis [10]. The R2R3-MYB transcription factor is the main MYB transcription activator in the MBW (MYB-bHLH-WD40) protein complex responsible for regulating anthocyanin biosynthesis, and the expression levels of R2R3-MYB transcription factors in various plants are highly positively correlated with anthocyanin content [43]. Recent studies have shown that the MYB transcription factor of the R2R3MYB type is also the main regulator of anthocyanin biosynthesis in potato [8]. We sent the control and low-temperature-treated tubers for sequencing during the period with the highest anthocyanin content. The sequencing results revealed that 48 MYB transcription factors were differentially expressed under low-temperature conditions. By analyzed the *StMYB* genes family, it was found that *StMYB113* and *StMYB308* transcription factors were significantly upregulated under low-temperature conditions and were clustered on a unified branch with Arabidopsis anthocyanin synthesis MYBs. Several studies have shown that *StMYB113* of the R2R3-MYB type can promote anthocyanin synthesis in potato tubers and tobacco leaves [27, 34], and R2R3-MYB lacking the conserved domain will exhibit loss of function and concomitant defects in anthocyanin accumulation [44, 45]. The R2 domain contains a conserved DNA-binding site. However, the *StMYB113* gene cloned from our material has only an R2 domain and is incomplete. Taken together, these findings and the results of transgenic tobacco experiments, we speculate that *StMYB113*, which contains this incomplete domain, can’t promote anthocyanin synthesis. After alignment, we found that the sequenced *StMYB308* gene had a complete domain similar to that of the *StAN2* gene, which has been reported to promote anthocyanin synthesis [27]. Compared with *StAN2*, *StMYB308* has 10 different protein translations at different positions, along with 7 more proteins at the end of the C-terminus and 13 missing after the R3 motif. Liu et al. reported that the presence of 10 amino acid motifs at the C-terminus is the best way to activate anthocyanin accumulation [34], and the R3 motif contains a conserved domain for MYB binding to bHLH proteins [46]. Therefore, we believe that *StMYB308* is an R2R3-MYB transcription factor whose specific function has not been reported. Among the 10 amino acid motifs of *StMYB113* and *StMYB308* we selected both exist at the C-terminus and aggregate with the Arabidopsis S6 family [33]. Therefore, it is speculated that *StMYB113* and *StMYB308* regulate anthocyanin synthesis.

Most of the activation domains of R2R3-MYB transcription factors are located at the N-terminus, and the repressive domains are located at the C-terminus [22]. Previous studies have shown that bHLH transcription factors can often act synergistically with MYB transcription factors to regulate anthocyanin accumulation. *StbHLH1* and *StJAF3* in potato have been shown to interact with potato MYB transcription factors to promote anthocyanin accumulation [34, 39, 47]. However, our transcriptome sequencing results showed that *StbHLH1* and *StJAF3* were expressed at extremely low levels, and there was no significant change in their expression in the low-temperature-treated tubers, presumably making it difficult to respond to low temperature and thereby affecting the *StMYB308*.

Under the CK and 15°C temperature treatments, the changes in anthocyanin content in the two pigmented potato tubers were positively correlated with the changes in *StMYB113* and *StMYB308* gene expression, and the subcellular localization indicated that both genes had nuclear transcriptional activation. Active nuclear transcription factor, thus allowing transient transformation of tobacco. As a result, *StMYB113* transgenic tobacco had no purple spots, and *StMYB308* transgenic tobacco has a significant increase in anthocyanin content, which promoted the upregulation of the endogenous bHLH genes *NtAn1a* and *NtAn1b*. As regulatory factors that transcriptionally activate the flavonoid pathway, *NtAn1a* and *NtAn1b* are strongly upregulated in overexpressed tobacco leaves and have been proven to promote anthocyanin synthesis, suggesting that *StMYB308* can interact with *NtAn1a* and *NtAn1b* to regulate anthocyanin synthesis. [23, 48, 49]. We hypothesized that *StMYB308* is involved in the regulation of anthocyanin biosynthesis in colored potatoes. It is speculated that *StMYB113* , which lacks a complete structural domain does not regulate anthocyanin synthesis and that the function of *StMYB308* can further promote the accumulation of anthocyanins.

## Conclusion

In this research, the Yunnan local characteristic pigmented potato varieties Jianchuanhong and Huaxinyangyu were used as test materials to explore the key transcription MYB factors that affect anthocyanin synthesis in pigmented potato tubers. *StMYB113* and *StMYB308* have low-temperature response functions and are significantly positively correlated with anthocyanin content. The two MYB transcription factors *StMYB113* and *StMYB308* were subsequently screened and analyzed by via bioinformatics, which revealed that *StMYB113* has only one MYB domain and that *StMYB308* has two MYB domains, which are R2R3-MYB transcription factors. The *StMYB113* and *StMYB308* overexpression genes were subsequently transferred into the leaves of safflower Dajinyuan. It was observed that the leaves overexpressing *StMYB308* exhibited obvious pigment accumulation.

The results showed that *StMYB308* is a transcription factor affecting potato anthocyanin biosynthesis, providing a theoretical reference for further study of the mechanism of the *StMYB113* and *StMYB308* transcription factors in potato anthocyanin synthesis. Future work may focus on the synergistic effect of *StMYBs* transcription factors and genes such as StbHLHs on anthocyanin synthesis.

## Materials and methods

### Materials

#### Plant material

The tissue culture–generated seedlings of the Yunnan local characteristic potato varieties—Jianchuanhong (red skin and red ring) and Huaxinyangyu (purple skin and purple ring)—were cultivated for 20 days, plants with uniform growth were collected, and the roots of the tissue culture–generated seedlings were washed with tap water. The base was subsequently transplanted into high-temperature sterilized substrate soil, which was subsequently placed in an artificial climate box to harden seedlings for 10 days. After 10 days of hardening, plants exhibiting uniform growth were selected, planted in nutrient pots and then treated at different temperatures until the end of the growth period. The day/night plants in the temperatures of the three temperature treatments were CK (20°C), 15°C, and 10°C, the photoperiod was 12 h/12 h, and the light intensity was 11,000 lx. The water and fertilizer management conditions were the same among the three treatments. Tubers were collected at 48 days, 72 days and 96 days after treatment, quick-frozen in liquid nitrogen and stored in a -80°C freezer. Three biological replicates were designed for each treatment.

*Nicotiana benthamiana* and Nicotiana safflower palnts were cultivated in an artificial climate box (25 ± 2℃) for four weeks for subcellular localization and transient transformation experiments.

None of the species used in this study were endangered or protected; all the plants were grown in greenhouses, and all the experiments on these plants complied with all the relevant guidelines and regulations. All the plant materials used were provided by Yunnan Agricultural University.

#### Database search for MYB proteins in Solanum and Arabidopsis

Due to the small and colorless tubers at 10℃, CK and 15℃ treated tubers were selected for sequencing. The pigmented potatoes materials sent for transcriptome sequencing were the tubers of pigmented potatoes with the highest anthocyanin content during the period [50]. The tubers from three plants were chopped and mixed together, with three biological replicates.

The names of the genes were based on the transcriptome annotation results.

Based on transcriptome sequencing, we obtained the cDNA and protein sequences of *StMYB113* and *StMYB308*. The relevant login numbers and sequences are listed in Additional file 1.

The corresponding *Arabidopsis thaliana* MYB protein sequences were downloaded from The Arabidopsis Information Resource (TAIR; http://www.Arabidopsis.org/).

The genome annotation sequence of potato (*Solanum tuberosum L.*) was obtained from the online data resources, https://solanaceae.plantbiology.msu.edu/pgsc_download.shtml.

### Main reagents

RNA extraction reagents, cDNA inversion kits, high-fidelity TA cloning kits, gel recovery kits and plasmid extraction kits were purchased from Tiangen Company (Beijing, China). The *Escherichia coli* strain DH5α and *Agrobacterium tumefaciens* strain GV3103-P19 were purchased from Kunming Tolu Biotechnology Co., Ltd.

### Carrier

The overexpression vector pCAMBIA2305.1 and the subcellular localization vector pHELLSGATE were provided by our laboratory.

## Experimental methods

### Analysis of the potato *StMYBs* family

The chromosomal information of the potato MYB family and the specific chromosomal location information were obtained from the transcriptome database. The chromosomal location map was drawn using the default parameters of the online software Map Gene to Chromosome (http://mg2c.iask.in/mg2c_v2.0/). To identify the conserved motif features of the potato StMYBs family, the amino acid sequences of all StMYB family members were uploaded to the online software MEME (http://meme-suite.org/tools/meme), the maximum number of motifs was set to 10, and the other parameters were set to default values. Finally, the TBtools tool was used to visualize the gene structure and conserved motifs according to the order shown in the potato StMYB family phylogenetic tree. With the help of MEGA X64 software, a phylogenetic tree of the potato StMYB family and Arabidopsis AtMYB transcription factors was constructed according to category [51]. R language 4.3.2 was used to draw a volcano plot of the StMYBs family in potato to show gens differentially expressed under low-temperature treatment.

### Bioinformatics analysis of *StMYB113* and* StMYB308* genes

The tertiary structures of the proteins encoded by *StMYB113* and *StMYB308* were predicted using the SWISS-MODEL online software tool. The *StMYB113* and *StMYB308* promoter sequences were analyzed with the bioinformatics software PlantCARE to predict the presence of cis-elements; online analysis software was usedInterPro, and SMART was used to predict and analyze the protein domains encoded by the *StMYB113* and *StMYB308* genes. DNAMAN was used to perform multiple alignments of amino acid sequences, and MEGAX was used to construct phylogenetic trees. The STRING (https://cn.string-db.org/) website was used for protein‒protein interaction prediction of the *StMYB113* and *StMYB308* genes.

### RNA extraction and cDNA strand synthesis

The CK-treated and low-temperature-treated Jianchuanhong tubers stored at -80°C were transferred to a mortar precooled with liquid nitrogen and ground to powder. An appropriate amount of TRIZOL was added to the powder, mixed well, and then subjected to strict mixing. RNA was extracted according to the instructions of the RNA extraction reagent, and the integrity of the RNA was subsequently verified 1.5% agarose gel electrophoresis. The product RNA was stored at -80°C.

The RNA was stored at -80°C and used to synthesize the first strand of cDNA according to the Evo M-MLV Reverse Transcription Premix Kit and stored at -20°C.

### Fluorescence quantitative PCR analysis

According to the sequences of *StMYB113* and *StMYB308*cds obtained by transcriptome sequencing, the primers *StMYB113* qF/qR and *StMYB308* qF/qR were designed with Prime Premier 5.0 (Table 1), and the *StGAPDH* gene of potato was used as the internal reference gene (Table 1).

**Table 1:** Sequence information of the primers used in the experiment

Gene	Prima	Function
*StGAPDH-F*	ATGAAGGACTGGAGAGGTGG	Reference Gene
*StGAPDH-R*	GAAAATGCTTGACCTGCTGT	
*StMYB113-qF*	AGCTTAATCCTCAAGTGGAGCATCG	*StMYB113* Expression Detection
*StMYB113-qR*	CGACCTGGTAACAAACGGGCTATC	
*StMYB308-qF*	GTGGCATCTTGTTCCAACTAGAGC	*StMYB308* Expression Detection
*StMYB308-qR*	AAGTGACCATCGGTTGCCTAAGAG	
*StMYB113-*LF	ATGTGTAGGATTAAATCGATGTCG	*StMYB113* CDS Clone
*StMYB113-*LR	TTAATTAAGTAGATTCCATAAGTCAATATCAGT	
*StMYB308-*LF	ATGACTTCACATGTAATGATCATGAG	*StMYB308* CDS Clone
*StMYB308-*LR	TTAATTAAGTAGATTCCATATATCATCTAGAGCA	
*StMYB113-*TF	CGGGGGACTCTTGACACTAGT ATGTGTAGGATTAAAT	*StMYB113* Overexpression
*StMYB113-*TR	GTCACCAATTCACACTT AATTAAGTAGATTCCATAAG	
*StMYB308-*TF	CGGGGGACTCTTGACACTAGT ATGACTTCACATGTAA	*StMYB308* Overexpression
*StMYB308-*TR	GTCACCAATTCACACTT AATTAAGTAGATTCCATATA	
*StMYB113-*YF	TTGGAGAGGACACGC ATGTGTAGGATTAAAT	*StMYB113* Subcellular Localization
*StMYB113-*YR	CCCTTGCTCACCAT TTACAGACACTTCCCTCCATAT	
*StMYB308-*YF	TTGGAGAGGACACGC ATGACTTCACATGTAATGATCA	*StMYB308* Subcellular Localization
*StMYB308-*YR	CCCTTGCTCACCAT CGCTGCCGCCGCCGCCATTAAGT	
*NtGAPDH*-F	GGTGTCCACAGACTTCGTGG	Reference Gene
*NtGAPDH*-R	GACTCCTCACAGCAGCACCA	
*NtAN1a*-F	ACCATTCTCGAACACCGAAG	*NtAN1a* Expression Detection
*NtAN1a*-R	TGCTAGGGCACAATGTGAAG	
*NtAN1b*-F	CTTGAACACTTCTCAAACCGA	*NtAN1b* Expression Detection
*NtAN1b*-R	TGCTAGGGCACAATGTGAAG	

Using the purified cDNA product as a template, a 20 μl qPCR system was configured. After the reaction, the relative expression level was calculated by the 2^−△△CT^ method [52] according to the cycle threshold (CT value) of the obtained gene.

### Fluorescence quantitative PCR analysis

After the plants were divided by treatment period, R language 4.3.2 was used to analyze the correlation between anthocyanin content and the expression levels of the *StMYB113* and *StMYB308* genes.

### Gene expression analysis

According to the *StMYB113* and *StMYB308*cds sequences obtained by transcriptome sequencing, the full-length amplification primers *StMYB113* LF/LR and *StMYB308* LF/LR (Table 1) were designed with Prime Premier 5.0, and the cDNA extracted from the pigmented potato tubers was used as a template for PCR amplification. The reaction mixture was 50 μl in volume. The amplified products were detected by 1.5% agarose gel electrophoresis, the cDNA was recovered by a Tiangen-Common DNA Product Purification Kit, and the recovered products were stored at -20°C.

### Construction of the overexpression vector

Using the Tiangen-PLB zero background rapid cloning kit, the purified cDNA product was ligated into the pLB vector, and the ligated product was subsequently transformed into competent DH5α cells, which were subsequently cultured on LB medium supplemented with Amp^+^ resistance at 37°C for 12–16 h. A single positive colony was picked and sent to Beijing Qingke Biotechnology Co., Ltd. For sequencing to confirm the target fragment.

The pCAMBIA2305.1 plasmid was used as a homologous cloning vector. The homologous primers *StMYB113* TF/TR and *StMYB308* TF/TR (Table 1) were designed with Prime Premier 5.0, and the cloning vector was ligated with homologous primers. The plasmid pCAMBIA2305.1 was digested with Xba I and Kpn I high-fidelity enzymes to obtain a linearized vector. Using the Tiangen-EasyGeno single-fragment recombinant cloning kit, the linearized pCAMBIA2305.1 vector was ligated with the homologous product, and the ligated product was transformed into competent DH5α cells and cultured on LB medium supplemented with Kan^+^ resistance at 37°C for 12–16 h. A single positive colony was picked and subsequently sent to Beijing Qingke Biotechnology Co., Ltd., for sequencing to confirm the target fragment.

### Subcellular localization analysis

Based on the sequences of *StMYB113* and *StMYB308* obtained by transcriptome sequencing, Prime Premier 5.0 was used to design the gene subcellular localization primers *StMYB113* YF/YR and *StMYB308* YF/YR (Table 1).

Using the pHELLSGATE plasmid as a homologous cloning vector, the plasmid pHELLSGATE was digested with Xba I and Kpn I high-fidelity enzymes to obtain a linearized vector. Using the Tiangen-EasyGeno single-fragment recombinant cloning kit, the linearized pHELLSGATE vector was ligated with the homologous product, and the ligated product was transformed into competent DH5α cells, which were subsequently cultured at 37°C for 12–16 h on LB medium supplemented with Kan^+^ resistance. The positive single colony was sent to Beijing Qingke Biotechnology Co., Ltd. for sequencing to confirm the target fragment. The constructed yellow fluorescent protein transient fusion expression vectors *StMYB113*-GFP and *StMYB308*-GFP were transformed into Agrobacterium-competent GV3101, which were subsequently cultured at 28°C for 48–72 h on YEB medium supplemented with Kan^+^ resistance. Afterwards, single colonies were picked and planted in 3 mL of liquid Incubate in YEB for 12 h. Then, 600 μl of the bacterial solution was transferred to 30 mL of liquid YEB, which was subsequently cultivated until the OD600 was approximately 0.6–0.8, collected and suspended by centrifugation. Tobacco buffer was added to adjust the OD600 to approximately 1.0, and the mixture was allowed to stand for 3 h at room temperature [53]. As a control, four-week-old *N. benthamiana* leaves were injected, and the fluorescence signals of the leaves were observed under a microscope after 8 days to confirm the subcellular localization of *StMYB113*-GFP and *StMYB308*-GFP.

### Transient transformation expression analysis of tobacco

The homologous cloned product was subsequently transformed into Agrobacterium-competent GV3101-P19, which were subsequently cultured on YEB medium supplemented with Kan^+^ resistance for 48–72 h at 28°C, and positive single colonies were picked and cultured in 3 mL of liquid YEB for 12 h. The following steps were the same as those used for GFP. Subsequently, four-week-old safflower Dajinyuan tobacco leaves were injected, and purple spots were observed on the leaves [53].

### RNA extraction and cDNA synthesis from tobacco leaves

The method was the same as that for RNA extraction and cDNA strand synthesis.

### Fluorescence-based quantitative PCR analysis of the transgenic tobacco

The *NtGAPDH* gene of tobacco was used as the internal reference gene for designing primers for tobacco anthocyanin-related structural genes (Table 1). The rest of the methods were the same as those used for fluorescence quantitative PCR analysis.

### Determination of transgenic tobacco anthocyanin contents

The anthocyanin content was determined by the pH differential spectrophotometry method described by Liu et al [28]. Weighing 0.5 g of fresh tobacco sample, adding 10 mL of anthocyanin extract (95% ethanol mixed with 1.5 mol/L hydrochloric acid) and grinding until homogenization. Afterwards, the mixture was subjected to ultrasonic treatment and centrifugation to extract the supernatant; 5 mL of anthocyanin extract was then added to the filter residue, which was subjected to ultrasonic treatment followed by centrifugation to extract the supernatant. The absorbance values of the extracts were measured at visible-spectrum absorption wavelengths of λ_max_ and λ_700_.

### Supplementary Information

**Additional file 1.**

**Additional** **file 2.**

**Additional file 3.**

## Data Availability

All raw transcriptomics sequencing data are being uploaded to the National Center for Biotechnology Information (https://www.ncbi.nlm.nih.gov/). BioProject: PRJNA978359.
