# Supplementary material for: Screening and functional analysis of StMYB transcription factors in pigmented potato under low-temperature treatment
Source: BMC Genomics. 2024 Mar 18;25:283. doi: 10.1186/s12864-024-10059-x (PMC10946176; doi:10.1186/s12864-024-10059-x)
Supplement: Supplementary file 1 — Additional file 1. [file 12864_2024_10059_MOESM1_ESM.docx]

Transcription factor sequences cloned in the experiment.

>*StMYB113* CDS [PGSC0003DMG400013966](https://ensembl.gramene.org/Solanum_tuberosum/Gene/Summary?db=core;g=PGSC0003DMG400013966;r=3:60532437-60534392;tl=DehjAbuOsHg7Rwa0-88517-10404405)

ATGTGTGGATTAAATCGATGTCGAAAGAGTTGCAGACTAAGGTGGCTAAATTATCTAAGGCCACATATCAAGAGAGGTGACTTTGCTCCAGATGAAGTGGATCTCATCTTGAGACTTCATAAACTCTTAGGCAATAGGTGGTCACTTATTGCTGGTAGACTTCCAGGAAGGACAGCAAATGATGTGAAGAACTATTGGAACACTCACTTTCAAAAGAAGTTAAATATTATTGCTCCTCCTCCTCCTCCTCGCCCTCGTCCTAATCATCATCTACAGATTAAGCATAAGAGCATCACGGTTAATAAGAATGAAATAATAAGACCTCAACCTCGGAACTTCTCAAACGTTAAGAAGAATAATTCTCATTGGTGCAACAACAAAAGTATGATCACAAACACATTAGACAAAGACGACAAACGTTGCAAGGAAATCGTAGTAAATATTTCTGAGAAGCCAACAGGAGAAAATACATCGTCGATAGACGATGGAGTTCAATGGTGGACAAATTTACTGGAAAATTGCAATGAAATTGAAGAAGAAGTAGCTGTTACAAATTTTGAAAAAACACCAACAATGTTGTTACATGAGGAAATATCACCACCGTTAATTAATGGTGAAGGCAATTCCATGCAACAAGGACAAAGTCATGATTGGGATGACTTTTCAACTGATATTGACTTATGGAATCTACTTAATTAA

>*StMYB113* Protein [PGSC0003DMT400036284](https://ensembl.gramene.org/Solanum_tuberosum/Transcript/ProteinSummary?db=core;t=PGSC0003DMT400036284;tl=dDEMBJxk3SRgdNa2-88542-10404861)

MCRINRCRKSCRLRWLNYLRPHIKRGDFAPDEVDLILRLHKLLGNRWSLIAGRLPGRTANDVKNYWNTHFQKKLNIIAPPPPPRPRPNHHLQIKHKSITVNKNEIIRPQPRNFSNVKKNNSHWCNNKSMITNTLDKDDKRCKEIVVNISEKPTGENTSSIDDGVQWWTNLLENCNEIEEEVAVTNFEKTPTMLLHEEISPPLINGEGNSMQQGQSHDWDDFSTDIDLWNLL

>*StMYB308* CDS PGSC0003DMG400013965

ATGACTTCACATGTAATGATCATGAGTACTCCTATGATGTGTACATTTTTGGGAGTAATAAGGAAAGGTTCATGGACTGAAGAAGAAGATATTCTTTTGAGGAAATGTATTGATAAGTATGGAGAAGGAAAGTGGCATCTTGTTCCAACTAGAGCTGGATTAAACAGATGCAGAAAAAGTTGTAGACTGAGGTGGCTAAATTATCTAAGGCCACATATCAAGAGAGGTGACTTTGAACCAGATGAAGTGGATCTCATCTTGAGACTTCATAAGCTCTTAGGCAACCGATGGTCACTTATTGCTGGTAGACTTCCAGGAAGGACAGCTAACGATGTGAAAAACTATTGGAACACTAACCTTCTAAGGAAGCTAAATACTAGTACTAAATTTGCTCCTCAACCACAAGAAGGAATTAATACTAGTACTATTGCTCCTCAACCACAAGAAGGAATTAAGTATGGGCAAGCCAATGCCATAATAAGACCTCAACCTCAGAAATTCACAAGCTCCATGAAGATTAATGTCTCTTGGTGCAACAACAATAGTATGGTAAATAATGAAGAAGCATCGAAAGACAACAACGATATGCAATGGTGGGCAAATATACTGGAAAACTGCAATGACATTGGAGAAGGAGAAGCTGAAAGAACACTACCTTCATGTAAGGAAATTAATTGCAATGAAATTGATAAAGCACCAAGTTTGTTACATGAGGGAGGCAACTCCATGCAACAAGGACAAGGTGATGGTGGTTGGGATGAATTTGCTCTAGATGATATATGGAATCTACTTAATTAA

>*StMYB308* Protein [PGSC0003DMT400036283](https://ensembl.gramene.org/Solanum_tuberosum/Transcript/ProteinSummary?db=core;g=PGSC0003DMG400002515;r=3:60532437-60534392;t=PGSC0003DMT400036283;tl=8YsM0fzwUQrKHPBO-88520-10404545)

MTSHVMIMSTPMMCTFLGVIRKGSWTEEEDILLRKCIDKYGEGKWHLVPTRAGLNRCRKSCRLRWLNYLRPHIKRGDFEPDEVDLILRLHKLLGNRWSLIAGRLPGRTANDVKNYWNTNLLRKLNTSTKFAPQPQEGINTSTIAPQPQEGIKYGQANAIIRPQPQKFTSSMKINVSWCNNNSMVNNEEASKDNNDMQWWANILENCNDIGEGEAERTLPSCKEINCNEIDKAPSLLHEGGNSMQQGQGDGGWDEFALDDIWNLLN
